# Rebamipide as an Adjunctive Therapy for Gastrointestinal Diseases: An Umbrella Review

**DOI:** 10.3390/ph19010144

**Published:** 2026-01-14

**Authors:** Igor V. Maev, Alsu R. Khurmatullina, Dmitrii N. Andreev, Andrew V. Zaborovsky, Yury A. Kucheryavyy, Philipp S. Sokolov, Petr A. Beliy

**Affiliations:** 1 Department of Internal Disease Propaedeutics and Gastroenterology, Russian University of Medicine, 127473 Moscow, Russia; 2 Department of Pharmacology, Russian University of Medicine, 127473 Moscow, Russia; 3 Ilyinskaya Hospital, 143421 Krasnogorsk, Russia

**Keywords:** dyspepsia, endoscopic submucosal dissection, gastroprotection, helicobacter pylori, meta-analysis, NSAID-induced gastropathy, rebamipide, umbrella review

## Abstract

**Objective**: This umbrella review aimed to synthesize evidence from meta-analyses on the efficacy of rebamipide in major gastrointestinal disorders and dyspeptic symptoms. **Methods**: This umbrella review followed Joanna Briggs Institute standards and was registered in PROSPERO (CRD420251185686). A comprehensive search of MEDLINE, EMBASE, Cochrane, and Scopus (1 January 1985, to 10 September 2025) was conducted to identify systematic reviews and meta-analyses assessing rebamipide therapy. Methodological quality was appraised using AMSTAR-2, ROBIS, and GRADE tools. Pooled data were analyzed using fixed- or random-effects models according to heterogeneity, as assessed using the I^2^ statistic. **Results**: Eleven meta-analyses (88 primary studies) were included. Rebamipide significantly improved *H. pylori* eradication (OR = 1.76; 95% CI: 1.44–2.16), reduced NSAID-induced mucosal injury (OR = 2.72; 95% CI: 1.89–5.14), enhanced ulcer healing after endoscopic submucosal dissection (OR = 2.28; 95% CI: 1.42–3.65), and alleviated dyspeptic symptoms (OR = 2.95; 95% CI: 1.04–8.37). Overall evidence quality was moderate to high, with low to moderate risk of bias. **Conclusions**: Rebamipide demonstrates consistent therapeutic benefits across diverse gastrointestinal disorders, improving *H. pylori* eradication rates, mucosal protection, ulcer healing, and symptom relief. These findings support rebamipide as an effective and well-tolerated adjunctive agent for the prevention and management of upper gastrointestinal diseases.

## 1. Introduction

Gastrointestinal disorders, including chronic gastritis, peptic ulcers, non-steroidal anti-inflammatory drug (NSAID)-induced gastropathy, and complications following endoscopic submucosal dissection (ESD), are prevalent worldwide and contribute substantially to morbidity and mortality. In addition, some of these conditions are associated with an increased risk of cancer [1,2,3,4,5]. For instance, *Helicobacter pylori* (*H. pylori*)-associated gastritis can progress to gastric cancer in 1–3% of patients [6]. Early-stage lesions of gastric cancer are often treated by ESD, particularly in Japan and other Asian countries [7,8], but this procedure raises the risk of subsequent ulcers and bleeding [9]. NSAID-induced gastropathy is frequently associated with gastrointestinal bleeding, adding further burden to patient outcomes and healthcare systems [10].

Given the high prevalence and clinical impact of these gastrointestinal disorders, there is an increasing need for effective therapeutic strategies that both prevent mucosal injury and promote healing. Rebamipide is a gastroprotective agent, chemically known as 2-[(4-chlorophenyl)formamido]-3-(2-oxo-1,2-dihydroquinolin-4-yl)propanoic acid, widely used in Japan, South Korea, Russia, and other countries [11]. It has gained attention for its mucosal protective properties and its potential therapeutic benefits across the gastrointestinal conditions mentioned above.

A meta-analysis of 11 randomized controlled trials involving 1227 patients found that adding rebamipide to *H. pylori* eradication regimens significantly improved eradication success (OR 1.75, 95% CI: 1.31–2.33) [12]. Another study, including 13 randomized controlled trials, demonstrated that rebamipide significantly reduced the incidence of NSAID-induced gastrointestinal mucosal breaks compared to placebo (RR 0.55, 95% CI: 0.31–0.99) [13]. Regarding *H. pylori* eradication. Pooling data from six randomized controlled trials involving 724 patients showed that combining rebamipide with proton pump inhibitors (PPIs) promotes faster healing of ulcers following ESD (OR 2.40, 95% CI: 1.68–3.44) [14]. These findings demonstrate the clinical relevance of rebamipide in several gastrointestinal conditions. *H. pylori*-associated gastritis can progress to gastric cancer if untreated. ESD may result in mucosal injury and related complications. NSAID-induced mucosal injury remains a major cause of gastrointestinal bleeding and hospitalization. Together, these conditions pose an increasing therapeutic challenge in modern gastroenterology.

The magnitude of rebamipide therapeutic benefit appears to vary depending on the clinical context, as well as the geographical and ethnic populations in which trials were conducted—most notably, in the majority of studies from East Asian cohorts, prescribing practices, baseline mucosal pathology, and genetic profiles influencing drug metabolism differ from Western populations [15,16]. Therefore, although individual meta-analyses suggest that rebamipide confers clinical benefits in mucosal protection, ulcer healing, and *H. pylori* eradication enhancement, overall evidence validity remains limited by these sources of heterogeneity. Although several meta-analyses have evaluated rebamipide in these individual contexts, no umbrella review has yet synthesized this body of evidence.

This umbrella review aims to synthesize evidence from existing meta-analyses on the efficacy of rebamipide in preventing and managing gastrointestinal disorders.

## 2. Results

### 2.1. Study Selection

**Figure 1:**
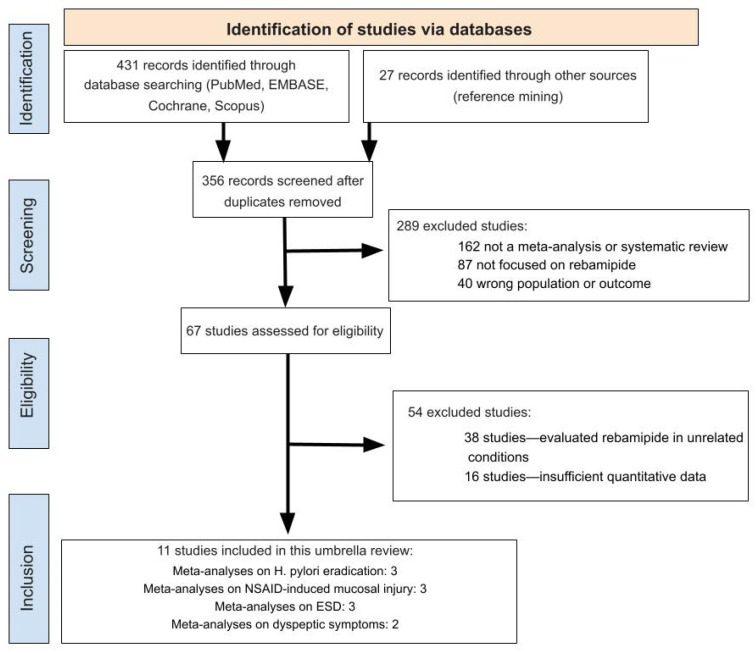
PRISMA (Preferred Reporting Items for Systematic Reviews and Meta-Analyses) flowchart.

A total of 458 records were retrieved through electronic database searches. After removing 102 duplicates, 356 unique records were screened by title and abstract. Of these, 289 were excluded because they were not meta-analyses, did not investigate rebamipide, or were otherwise irrelevant to the research question. The full texts of the remaining 67 articles were assessed for eligibility, leading to the exclusion of 54 studies for the following reasons: unrelated gastrointestinal conditions (*n* = 38), insufficient quantitative data for meta-analysis (*n* = 16). Ultimately, 11 meta-analyses met the inclusion criteria and were incorporated into the umbrella review: three on *Helicobacter pylori*, three on NSAID-induced mucosal injury, three on outcomes following ESD, and two on dyspeptic symptoms (Figure 1). Table 1 summarizes the characteristics of included studies.

### 2.2. ROBIS Assessment

Supplementary Section S1 presents an overview of the risk of bias assessment performed using the ROBIS tool. In evaluating the ESD-induced ulcer healing and rebamipide use, the highest risk of bias was identified in the Data collection and study appraisal domain, whereas the lowest risk of bias was observed in the Study eligibility criteria and Identification and selection of studies domains. In the case of *H. pylori* eradication and rebamipide use, data collection and study appraisal again emerged as the main source of bias, whereas the Synthesis and findings domain demonstrated the lowest level of bias.

### 2.3. GROOVE Analysis

Across the included systematic reviews and meta-analyses, 88 studies were identified, encompassing a total of 7596 participants (most of the studies were conducted in Japan, Republic of Korea and Russia). To evaluate the degree of evidence overlap, three separate GROOVE analyses were performed. For *H. pylori* eradication and rebamipide use, the corrected covered area (CCA) was 32.14%, reflecting a high degree of overlap; after accounting for chronological structural missingness, the adjusted CCA remained within the same range. In the case of ESD-induced ulcer healing and rebamipide use, the overlap proved to be more pronounced, with a CCA of 42.86%, which similarly persisted following adjustment. For NSAID-induced ulcers and rebamipide use, the GROOVE analysis demonstrated a CCA of 20.45%, indicating an intermediate level of redundancy among the three domains. Finally, for dyspeptic symptoms treatment CCA was 0%, which is the lowest among all studies.

Graphical visualizations of the GROOVE findings for each analysis are presented in Supplementary Section S2.

## 3. Effectiveness of Rebamipide Supplementation

### 3.1. H. pylori Eradication

**Figure 2:**
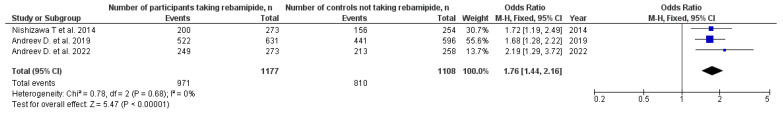
Odds ratio for *H. pylori* eradication rates in regimens including rebamipide versus standard therapy [12,17,18].

The pooled analysis demonstrated a statistically significant benefit of adding rebamipide to standard eradication regimens for *H. pylori*, with OR of 1.76 (95% CI: 1.44–2.16). No heterogeneity was observed across the included studies (I^2^ = 0%), indicating consistency of results, so the fixed effects model was applied. Figure 2 illustrates the pooled odds ratios (ORs) for eradication outcomes in regimens with and without rebamipide, highlighting the superiority of combination therapy The pooled eradication rate was 82.94% (95% CI: 73.09–90.91) in the rebamipide group, compared to 73.03% (95% CI: 61.96–82.82) in the control group.

**Figure 3:**
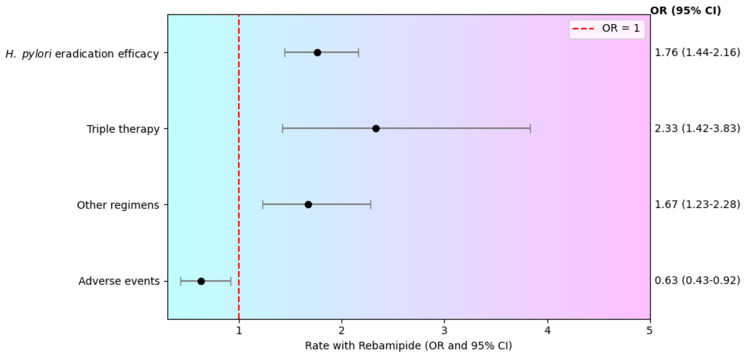
Forest Plot of Rebamipide Adjunct Therapy in *H. pylori* Eradication.

A subgroup data resynthesis was performed using aggregated data from 3 published meta-analysis evaluating the effect of rebamipide as part of *H. pylori* eradication therapy. When analyzed by treatment scheme, the pooled OR for triple therapy regimens including rebamipide was 2.33 (95% CI: 1.42–3.83). The pooled eradication rate for patients receiving triple therapy plus rebamipide was 90.54% (95% CI: 86.97–93.40), compared with 82.05% (95% CI: 77.17–86.27) in the control group. For other combination regimens, the effect remained significant, with an OR of 1.67 (95% CI: 1.23–2.28); the pooled eradication rate for patients in the rebamipide group was 77.99% (95% CI: 67.56–86.88), while in the control group it was 65.33% (95% CI: 51.45–78.00). The incidence of adverse events differed significantly between the rebamipide and control groups (OR = 0.63; 95% CI: 0.43–0.92), indicating that the addition of rebamipide was associated with a reduced risk of treatment-related toxicity. The pooled adverse effect rate for patients receiving rebamipide was 21.08% (95% CI: 6.80–40.56), compared with 24.57% (95% CI: 16.11–34.17) in the control group. The findings are presented in Figure 3.

### 3.2. NSAID-Induced Mucosal Injury

**Figure 4:**
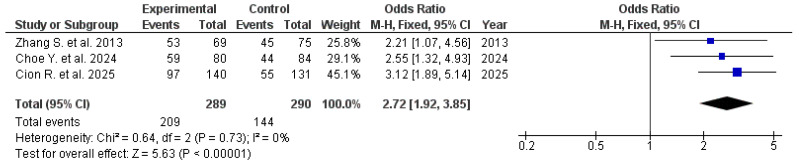
Effect of Therapy in Patients Receiving Rebamipide vs. Controls During NSAID Therapy [13,19,20].

We assessed the effect of rebamipide in patients receiving NSAIDs compared with control groups. Because the heterogeneity among the included studies was minimal (I^2^ = 0%), a fixed-effect model was used for the analysis. The combined results indicated that rebamipide administration markedly enhanced clinical outcomes relative to placebo therapy, yielding an overall OR of 2.72 (95% CI: 1.89–5.14; Figure 4). The therapeutic effect was achieved in 72.14% (95% CI: 66.87–77.13) of the rebamipide group, compared to 55.48% (95% CI: 49.59–61.26) of the control group.

In addition, separate subgroup analyses for gastric and small-bowel pathology confirmed the robustness of the overall findings. Rebamipide showed a significant therapeutic advantage in patients with gastric lesions, yielding an OR of 2.44 (95% CI: 1.19–5.01), while a similarly favorable effect was observed in small-bowel injury, with an OR of 2.79 (95% CI: 1.47–5.32).

In the subgroup analysis focused on NSAID-induced gastrointestinal injury, the addition of rebamipide to PPI therapy was assessed in only one available meta-analysis [13]. The authors reported that this combination showed a tendency toward greater efficacy compared with PPI monotherapy; however, the difference did not reach statistical significance. The reported effect size was OR 1.74 (95% CI: 0.88–3.44), indicating a trend favoring the addition of rebamipide but with the confidence interval crossing 1, thus showing no definitive evidence of superiority.

### 3.3. ESD-Induced Ulcer Healing

**Figure 5:**
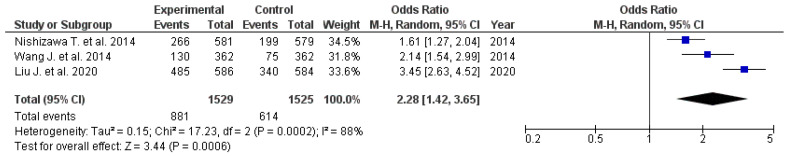
Odds ratio for the effect of adding rebamipide to PPI therapy on ESD-induced ulcer healing [14,21,22].

A meta-analysis was conducted to evaluate the impact of adding rebamipide to PPI therapy on ESD-induced ulcer healing. Owing to substantial heterogeneity among the included studies (I^2^ = 88%), a random-effects model was applied. The pooled estimate demonstrated that rebamipide supplementation significantly improved healing outcomes, with an OR of 2.28 (95% CI: 1.42–3.65). Figure 5 presents the pooled ORs for treatment with and without rebamipide, highlighting the beneficial effect of combination therapy. The ulcer healing rate was 55.75% (95% CI: 26.47–82.98) in the PPI + rebamipide group, compared to 37.25% (95% CI: 17.86–59.07) in the control group (PPI alone).

### 3.4. Management of Dyspeptic Symptoms

**Figure 6:**
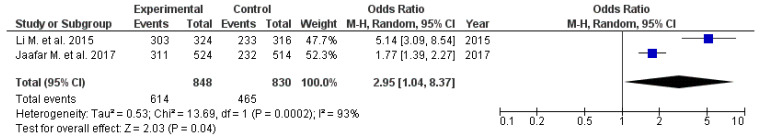
Odds ratio for the effect of adding rebamipide to dyspeptic symptoms treatment [24,25].

The analysis evaluating the adjunctive use of rebamipide for the management of dyspeptic symptoms revealed a significant overall effect. Owing to considerable heterogeneity among the included studies (I^2^ = 93%), data were analyzed using a random-effects model. The pooled OR was 2.95 (95% CI: 1.04–8.37), indicating that patients receiving rebamipide in addition to standard therapy were more likely to experience symptom relief than those in the control group. Figure 6 presents the findings. Although variability across studies was high, the general trend favored the addition of rebamipide as a beneficial component of dyspepsia treatment. In rebamipide group 78.99% (95% CI: 38.74–99.72) of patients achieved therapeutic effect, while in control group only 59.78% (95% CI: 31.38–84.99) managed to attain the relief of symptoms.

## 4. Discussion

**Figure 7:**
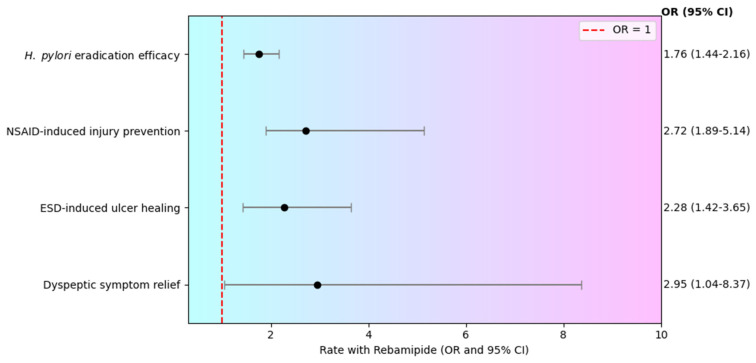
Overview of the clinical effectiveness of rebamipide across four major gastrointestinal conditions.

This umbrella review comprehensively synthesized evidence from existing meta-analyses to evaluate the clinical effectiveness of rebamipide across 4 major gastrointestinal conditions—*H. pylori* infection, NSAID-induced mucosal injury, complications following ESD and dyspeptic symptoms (Figure 7).

The pooled data demonstrate that rebamipide consistently confers significant protective and therapeutic benefits across these settings. Across the overall pool of included studies, rebamipide was most commonly administered at a dose of 100 mg three times daily, with treatment durations ranging from 1 to 8 weeks, depending on the clinical indication.

When added to standard *H. pylori* eradication regimens, rebamipide significantly improved eradication success (OR 1.76 (95% CI: 1.44–2.16)). This finding aligns closely with the meta-analysis by Andreev et al. (2019), which included the largest patient population to date and reported results consistent with our analysis [12]. In the context of *H. pylori*-associated gastritis, rebamipide exerts disease-specific effects by inhibiting bacterial adhesion to the gastric epithelium, suppressing *H. pylori*–induced inflammatory signaling (TNF-α, IL-8, NF-κB), and reducing urease-mediated mucosal toxicity [12,25]. Experimental data further suggest that rebamipide modulates β-catenin signaling pathways activated by cytotoxin-associated protein A (CagA), which may limit aberrant epithelial proliferation and inflammation associated with gastric carcinogenesis [26]. These findings underline the dual role of rebamipide in enhancing bacterial eradication and promoting mucosal restitution, which is critical in preventing *H. pylori*-associated carcinogenesis.

For NSAID-induced mucosal injury, rebamipide markedly reduced gastrointestinal damage (pooled OR 2.72, 95% CI: 1.89–5.14), underscoring its robust gastroprotective efficacy. In this case, the gastroprotective effects of rebamipide are closely related to its ability to scavenge reactive oxygen species, inhibit neutrophil adhesion via downregulation of CD11/CD18 integrins, and restore prostaglandin synthesis through COX-2 induction [16]. These mechanisms are crucial for reducing the incidence of NSAID-related erosions, bleeding, and ulcer formation—major contributors to hospitalization in patients on long-term anti-inflammatory therapy. Finally, recent pooled analyses demonstrate that rebamipide consistently confers significant gastroprotective effects across multiple indications, further supporting its use as an adjunct in patients at risk of mucosal injury or impaired healing [27]

Similarly, in patients undergoing ESD, rebamipide combined with PPIs substantially accelerated ulcer healing (pooled OR 2.28, 95% CI: 1.42–3.65). Rebamipide exerts multifaceted gastroprotective and cytoprotective effects that are central to maintaining gastrointestinal mucosal integrity [11]. Its actions involve activation of the ERK1/2 and p38 MAPK pathways, leading to COX-2 induction and increased prostaglandin E2 and prostacyclin production, which enhance mucosal defense and repair [28]. In parallel, rebamipide activates AMPK and acetyl-CoA carboxylase phosphorylation, shifting intracellular signaling toward the NRF2 anti-inflammatory pathway while suppressing NF-κB activation [16]. This modulation results in reduced IL-17 production and enhanced mucin gene expression (MUC1, MUC2, MUC4), thereby reinforcing mucosal barrier function [29,30,31]. The drug also restores epithelial tight junction integrity by upregulating ZO-1 and claudin-1 proteins and promotes angiogenesis through increased expression of VEGF, EGFR, and FGFR-2, which accelerates mucosal regeneration [32]. Following ESD, rebamipide promotes ulcer healing by enhancing angiogenesis and epithelial restitution [33]. Additionally, rebamipide’s effects on the Rho kinase pathway enhance epithelial restitution and wound closure in the post-ESD setting [28,34].

Patients with dyspepsia receiving rebamipide in addition to standard therapy had significantly greater odds of achieving symptom relief (OR 2.95; 95% CI 1.04–8.37) than those receiving standard treatment without rebamipide. Similarly, recent studies confirm its efficacy in improving dyspeptic symptoms of both organic and functional causes [35]. The drug supports gastric motility by stimulating prostaglandin production [11]. In functional dyspepsia, a condition characterized by pathophysiological disturbances such as delayed gastric emptying, visceral hypersensitivity, and increased duodenal epithelial permeability, rebamipide exerts a protective effect on the mucosal barrier [36]. Clinical data show that it can reduce markers of permeability—such as serum zonulin—and decrease inflammatory cell infiltration in the duodenum [37]. Collectively, these mechanisms contribute to symptom relief and improved quality of life in patients with both organic and functional dyspepsia.

Although meta-analyses for gastroesophageal reflux disease are lacking, clinical evidence suggests that rebamipide may also be effective in this context. Studies report improvements in mucosal integrity and symptomatic relief in patients with erosive and non-erosive gastroesophageal reflux disease, indicating a potential therapeutic benefit similar to that observed in *H. pylori* gastritis and NSAID-induced injury [38,39,40].

From a clinical perspective, rebamipide appears to offer the greatest therapeutic value as an adjunctive agent in patients with erosive or ulcerative lesions of the gastrointestinal mucosa, where enhanced epithelial protection and mucosal repair are required in addition to primary therapy. In particular, its use may be especially justified in comorbid patients receiving long-term NSAID therapy who have additional risk factors for mucosal injury, including *H. pylori* infection, advanced age, a history of peptic ulcer disease, or concomitant use of anticoagulants or glucocorticosteroids.

This umbrella review has some limitations. Most included meta-analyses were based on studies from East Asian populations, which may restrict the applicability of results to other regions. Some overlap among primary studies and residual heterogeneity, particularly in analyses of dyspeptic symptoms, may have influenced the pooled estimates. Despite these limitations, the study has notable strengths, including the comprehensive synthesis of evidence across multiple gastrointestinal conditions, rigorous quality and bias assessment using validated tools, quantitative evaluation of study overlap, and consistent findings supporting rebamipide’s therapeutic efficacy across different clinical contexts.

## 5. Materials and Methods

### 5.1. Search Strategy

We performed this umbrella review following the methodological standards of the Joanna Briggs Institute [41]. This approach is appropriate when multiple systematic reviews address related research questions, as it synthesizes their findings, identifies areas of agreement and divergence, and highlights remaining evidence gaps relevant to clinicians, policymakers, and researchers. Our methodology adhered to current best practices and was aligned with previously published umbrella reviews [42]. The review protocol was prospectively registered in the PROSPERO database (registration ID: CRD420251185686). A comprehensive literature search was conducted in line with the PRISMA 2020 reporting guidelines for systematic reviews and meta-analyses [43]. The completed PRISMA-P checklist can be found in the Supplementary Materials (Table S1).

To ensure comprehensive coverage, we conducted a systematic search across several databases, including MEDLINE/PubMed, EMBASE, Cochrane and Scopus. The search spanned the period from 1 January 1985 to 10 September 2025. Eligible meta-analyses were those that synthesized original studies on rebamipide in at least one of the three target gastrointestinal conditions. Reviews lacking pooled quantitative analyses or focusing on unrelated interventions were excluded from this assessment.

To identify all relevant evidence, we developed predefined search strategies tailored to each of the four target conditions: NSAID-induced mucosal injury, *H. pylori*-associated gastritis, complications following ESD, and dyspeptic symptoms.

For NSAID-induced mucosal injury, the following terms were used: (“Anti-Inflammatory Agents, Non-Steroidal”[MeSH] OR NSAID* OR “nonsteroidal anti-inflammatory drug*”) AND (“Peptic Ulcer”[MeSH] OR “Gastrointestinal Diseases”[MeSH] OR “Gastric ulcer” OR “Duodenal ulcer” OR “Gastrointestinal bleeding”) AND (“Rebamipide”[MeSH] OR Rebamipide).

For *H. pylori*-associated gastritis, the search strategy was: (“*Helicobacter pylori*”[MeSH] OR “*H. pylori*”) AND (“Gastritis”[MeSH] OR gastritis OR “gastric mucosa”) AND (“Rebamipide”[MeSH] OR Rebamipide).

For complications following ESD, the search string was: (“endoscopic submucosal dissection” OR ESD) AND (“Postoperative Complications”[MeSH] OR “Gastrointestinal Hemorrhage”[MeSH] OR “Ulcer”[MeSH] OR complication* OR bleeding OR ulcer*) AND (“Rebamipide”[MeSH] OR Rebamipide).

Finally, for dyspeptic symptoms, the following terms were used: (“Dyspepsia”[MeSH] OR dyspepsia OR “dyspeptic symptoms” OR “functional dyspepsia”) AND (“Rebamipide”[MeSH] OR Rebamipide).

These search strategies were adapted as necessary for each database. Filters were applied to identify meta-analyses and systematic reviews; randomized controlled trials and observational studies were included only when part of eligible meta-analyses.

### 5.2. Eligibility Criteria and Quality Assessment

The methodological framework followed the PICO approach. The Population comprised patients with one of the three target gastrointestinal conditions: *H. pylori*, NSAID-induced mucosal injury, or adverse events following ESD. Only studies that explicitly analyzed human subjects were eligible; research conducted exclusively in animals or in vitro was excluded. The Intervention of interest was treatment with rebamipide, either as monotherapy or as part of a combined regimen. The Comparison group included placebo, standard medical therapy without rebamipide, or alternative pharmacological regimens. The Outcomes of interest were clinical efficacy measures (e.g., successful *H. pylori* eradication, prevention or healing of NSAID- or ESD-induced mucosal injury, relief of dyspeptic symptoms), reported effect sizes (odds ratios, relative risks), and indicators of heterogeneity and study quality. The Study design was restricted to systematic reviews with or without meta-analysis that synthesized randomized or non-randomized controlled clinical trials. Observational studies and other non-experimental designs were excluded. No language restrictions were applied.

Studies were excluded if they did not meet accepted methodological standards for systematic reviews. Studies focused exclusively on experimental laboratory data, without human clinical outcomes, were excluded. Additional exclusion criteria included insufficient methodological transparency, unclear outcome reporting, or the absence of a well-defined study population or intervention description.

Two independent reviewers (F.S.S. and Y.A.K.) evaluated the methodological quality of eligible systematic reviews and meta-analyses using a modified version of the AMSTAR-2 tool (A Measurement Tool to Assess Systematic Reviews) [44]. This instrument includes 16 items rated as “yes” (criterion fully met), “no” (criterion unmet or insufficient data provided), or “partial yes” (criterion partially fulfilled). Interrater reliability was assessed prior to consensus by calculating both Cohen’s kappa (κ) statistic and percentage agreement. κ values > 0.7 were interpreted as strong agreement, 0.5–0.7 as moderate, and <0.5 as weak reliability.

### 5.3. Risk of Bias Evaluation

The methodological quality of randomized controlled trials included in the systematic reviews was appraised with the ROBIS instrument (Revised Tool for Risk of Bias) [45]. This tool covers five domains: (1) Study eligibility criteria; (2) Identification and selection of studies; (3) Data collection and study appraisal; (4) Synthesis and findings. Each domain is structured around signaling questions that guide judgments, which are then categorized as “low risk,” “some concerns,” or “high risk.” Certainty of evidence for all evaluated associations was further graded using the updated GRADE framework (Grading of Recommendations Assessment, Development and Evaluation) [46].

Two reviewers (P.B.A. and A.Z.V.) independently performed the assessments, with disagreements resolved through discussion until consensus was reached. For visualization, we applied the ROBIS tool, which displays risk-of-bias ratings across domains and overall assessments in traffic-light style plots.

### 5.4. Overlap of Primary Studies

The extent of overlap in primary studies across systematic reviews was quantified using the GROOVE tool (Graphical Representation of Overlap for OVErviews) [47]. GROOVE constructs an evidence matrix, calculates the number of unique and overlapping studies, and reports the Corrected Covered Area (CCA) as a summary measure.

### 5.5. Data Extraction

Screening and data collection were carried out by two independent reviewers (A.R.K. and D.N.A.). The first stage involved evaluating titles, abstracts, and keywords for relevance. When consensus was not reached or abstracts lacked sufficient detail, the full text was retrieved. A second round of screening was based on full-text review to ensure eligibility criteria were satisfied. Data were then extracted following a standardized protocol.

From each review, we recorded the publication year, number of included primary studies, pathology under investigation, treatment regimens (rebamipide, comparators), population size, outcome definitions, number of effective cases, and the statistical model applied. Additionally, we extracted data on the lines of therapy, frequency of adverse events, and the overall effectiveness in *H. pylori* eradication.

Comparative effectiveness was extracted as relative risk (RR) and odds ratio (OR) with 95% confidence intervals.

### 5.6. Statistical Analysis

After data extraction, the primary outcomes reported in the original studies were recalculated using the odds ratio (OR) model. All efficacy outcomes were analyzed in favor of rebamipide, with an OR greater than 1 indicating improved therapeutic efficacy. Conversely, for safety outcomes (adverse events), an OR greater than 1 indicated an increased likelihood of adverse effects associated with rebamipide.

Given the heterogeneity in populations, interventions, and outcomes, a random-effects model was applied for synthesis where I^2^ was greater than 50%. Between-study heterogeneity was also quantified by the I^2^ statistic. To assess variability between meta-analyses (i.e., heterogeneity across different published meta-analyses included in this umbrella review), we compared pooled effect estimates and corresponding heterogeneity statistics (I^2^) reported for each outcome. We visually inspected the range of effect sizes and heterogeneity estimates across meta-analyses addressing the same clinical question, noting any substantial differences in magnitude, direction, or consistency of results. We also considered differences in study characteristics, populations, interventions, and outcome definitions as potential contributors to between-meta-analysis variability. Where appropriate, we discussed potential sources of between-meta-analysis heterogeneity and its implications for evidence certainty and interpretation.

Potential publication bias was investigated through funnel plots and Egger’s regression test (Supplementary Materials, Section S3). All results were expressed with 95% confidence intervals, and statistical significance was set at *p* < 0.05. Analyses were performed using RevMan software (version 5.4.1; London, UK).

## 6. Conclusions

This umbrella review demonstrates that adjuvant therapy with rebamipide is more effective than conventional treatment for several major gastrointestinal conditions. In *H. pylori* infection, rebamipide significantly improved eradication rates. For NSAID-induced injury, it substantially reduced the risk of mucosal damage, underscoring its broad mucosal protective effect and clinical versatility. In patients undergoing ESD, the combination of rebamipide with PPIs significantly accelerated ulcer healing. Collectively, these findings position rebamipide as an effective and well-tolerated adjunctive agent for preventing and managing diverse gastrointestinal disorders.

## Figures and Tables

**Table 1:** Key characteristics of the included systematic reviews and meta-analyses, outcome focus, sample size, intervention with rebamipide, comparator treatments, risk of bias assessments, and quality evaluations.

Comparison of *H. pylori* Eradication Rates With and Without Rebamipide								
Author, year	Types of included studies and number of included studies, *n*	Number of participants taking rebamipide, *n*	Number of participants achieving successful eradication with rebamipide, *n*	Number of controls not taking rebamipide, *n*	Number of participants achieving successful eradication without rebamipide, *n*	Effect Measure	Quality evaluation, AMSTAR-2	Quality evaluation, Grade
Nishizawa T. et al. 2014 [17]	6 RCTs	273	200	254	156	OR 1.74 (95% CI: 1.19–2.53)	Moderate	Low
Andreev D. et al. 2019 [12]	11 RCTs	631	522	596	441	OR 1.75 (95% CI: 1.31–2.34)	High	High
Andreev D. et al. 2022 [18]	6 RCTs	273	249	258	213	OR 2.16 (95% CI: 1.27–3.69)	Moderate	Moderate
**Effect of Therapy in Patients Receiving Rebamipide vs. Controls During NSAID Therapy**								
**Author, year**	**Types of included studies and number of included studies, *n***	**Number of participants taking rebamipide, *n***	**Number of patients without NSAID-induced mucosal injury, *n***	**Number of controls, *n***	**Number of controls without NSAID-induced mucosal injury, *n***	**Effect Measure**	**Quality evaluation, AMSTAR-2**	**Quality evaluation, GRADE**
Zhang S. et al. 2013 [19]	15 RCTs	69	53	75	45	RR 2.70 (95% CI: 1.02–7.16)	High	Moderate
Choe Y. 2024 [20]	5 RCTs	80	59	84	40	OR 2.55 (95% CI: 1.32–4.93)	High	Moderate
Cion R. et al. 2025 [13]	13 RCTs	140	97	131	55	OR 3.12 (95% CI: 1.89–5.14)	Moderate	Moderate
**Impact of Rebamipide Addition to PPI Therapy on ESD-Induced Ulcer Healing**								
**Author, year**	**Types of included studies and number of included studies, *n***	**Participants Receiving Rebamipide + PPI, *n***	**Pooled healing rates (Rebamipide + PPI), *n***	**Participants Receiving PPI Only, *n***	**Pooled healing rates (PPI Only), *n***	**Effect Measure**	**Quality evaluation, AMSTAR-2**	**Quality evaluation, GRADE**
Nishizawa T. et al. 2015 [21]	11 RCTs	581	266	579	199	OR 2.28 (95% CI: 1.57–3.31)	Moderate	Low
Wang J. et al. 2014 [14]	6 RCTs	362	130	362	75	OR 2.40 (95% CI: 1.68–3.44)	High	Moderate
Liu J. et al. 2020 [22]	9 RCTs	586	485	584	340	RR 1.42 (95% CI: 1.13–1.78)	High	High
**The Effect of Rebamipide on Dyspeptic Symptoms**								
**Author, year**	**Types of included studies and number of included studies, *n***	**Number of participants taking rebamipide, *n***	**Number of patients who had relieved symptoms, *n***	**Number of controls, *n***	**Number of controls who had relieved symptoms, *n***	**Effect Measure**	**Quality evaluation, AMSTAR-2**	**Quality evaluation, GRADE**
Li M. et al. 2015 [23]	12 RCTs	324	303	316	233	RR 1.23 (95% CI: 1.06–1.41)	Moderate	High
Jaafar M. et al. 2018 [24]	17 RCTs	524	311	514	232	OR 1.77 (95% CI: 1.39–2.27)	Low	Moderate

RCTs—Randomized Controlled Trials.

## Data Availability

No new data were created or analyzed in this study. Data sharing is not applicable to this article.

## Supplementary Materials

The following supporting information can be downloaded at: https://www.mdpi.com/article/10.3390/ph19010144/s1. Section S1. Completed PRISMA-P checklist. Section S2: ROBIS assessment; Section S3: Graphical visualizations of the GROOVE findings. Table S1. PRISMA Checklist of Items to Include When Reporting a Systematic Review Involving a Network Meta-analysis.
