# Supplementary material for: Rebamipide as an Adjunctive Therapy for Gastrointestinal Diseases: An Umbrella Review
Source: Pharmaceuticals (Basel). 2026 Jan 14;19(1):144. doi: 10.3390/ph19010144 (PMC12845245; doi:10.3390/ph19010144)
Supplement: Supplementary file 1 [file pharmaceuticals-19-00144-s001.zip › pharmaceuticals-4078943-supplementary.pdf]

Supplementary Section S1. ROBIS assessment

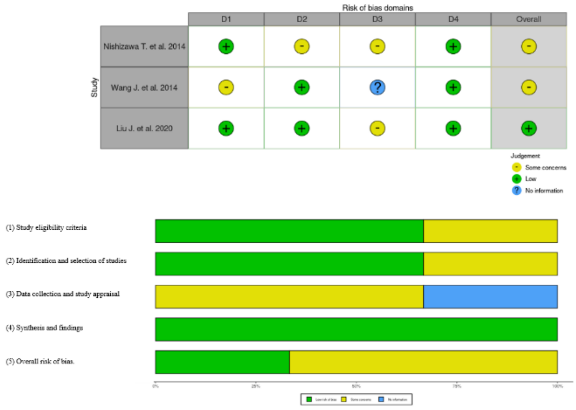

Figure S1. ROBIS assessment of ESD-induced ulcer healing and rebamipide use studies, [14], [21], [22].

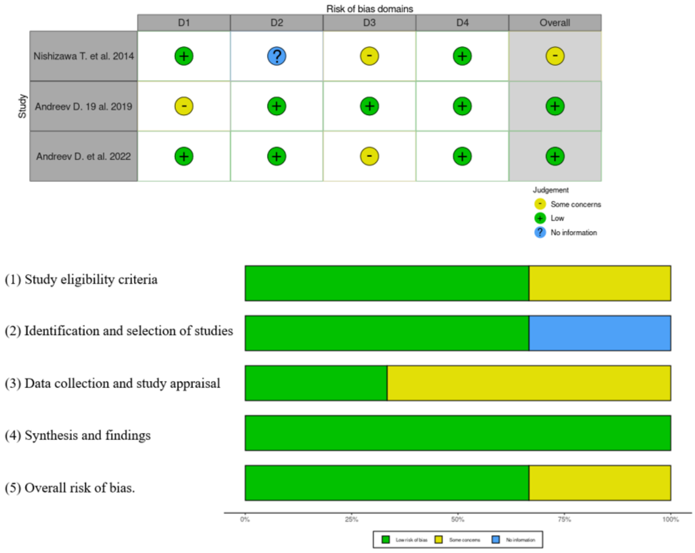

Figure S2. ROBIS assessment of *H. pylori* eradication and rebamipide use studies, [12], [17], [18]

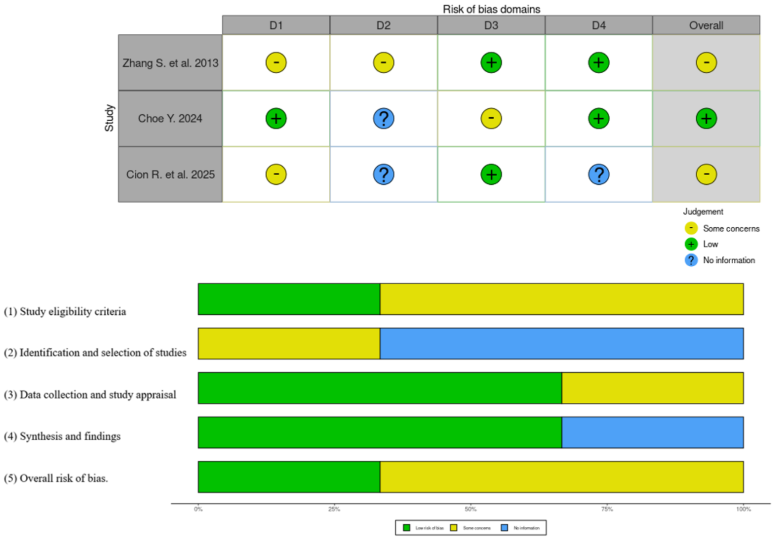

Figure S3. ROBIS assessment of NSAID and rebamipide use studies, [13], [19], [20]

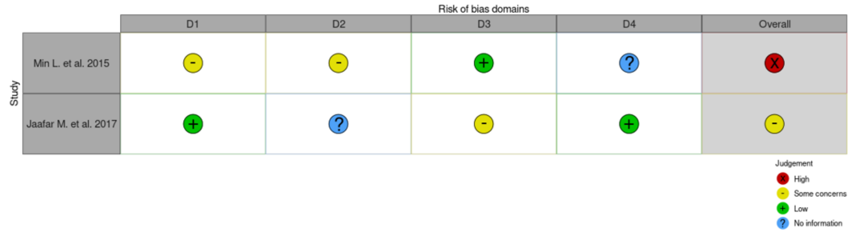

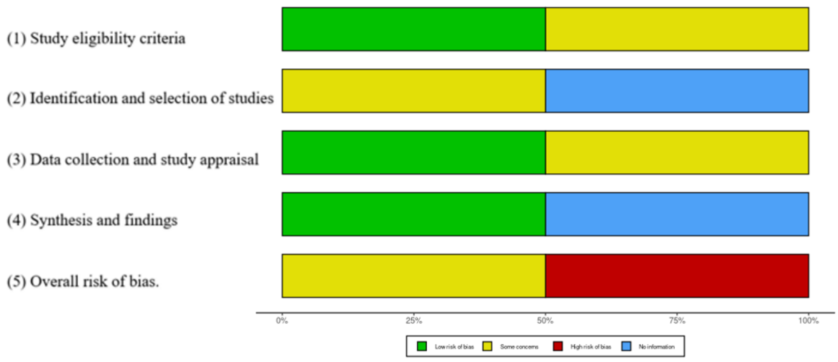

Figure S4. ROBIS assessment of dyspeptic symptoms and rebamipide use studies, [24], [25].

Supplementary Section S2. Graphical visualizations of the GROOVE findings

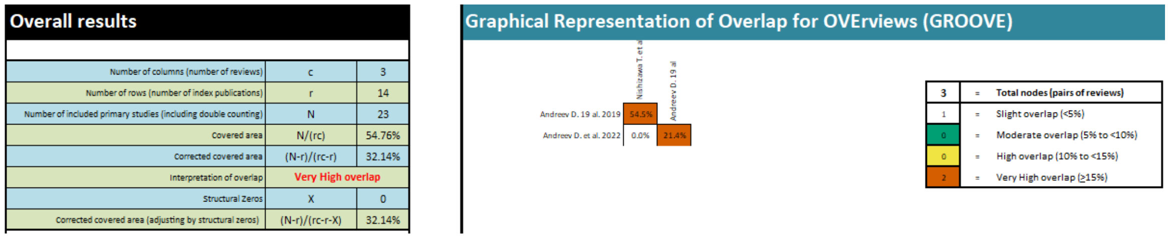

Overall results

Number of columns (number of reviews)	c	3
Number of rows (number of index publications)	r	14
Number of included primary studies (including double counting)	N	23
Covered area	N/(rc)	54.76%
Corrected covered area	(N-r)/(rc-r)	32.14%
Interpretation of overlap	Very High overlap	
Structural Zeros	X	0
Corrected covered area (adjusting by structural zeros)	(N-r)/(rc-r-X)	32.14%

Figure S5. GROOVE analysis of overlap in studies on *H. pylori* eradication and rebamipide use

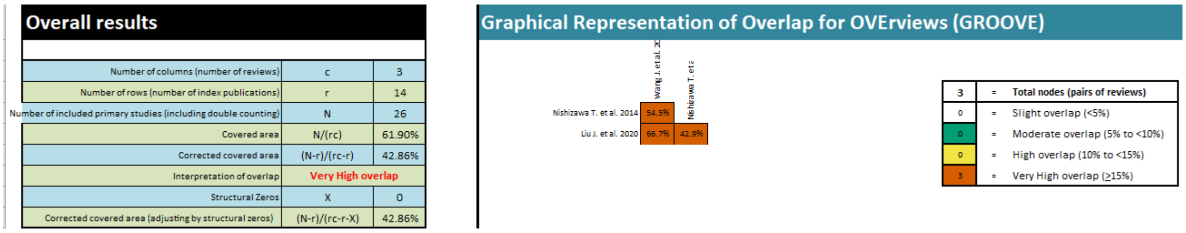

Overall results

Number of columns (number of reviews)	c	3
Number of rows (number of index publications)	r	14
Number of included primary studies (including double counting)	N	26
Covered area	N/(rc)	61.90%
Corrected covered area	(N-r)/(rc-r)	42.86%
Interpretation of overlap	Very High overlap	
Structural Zeros	X	0
Corrected covered area (adjusting by structural zeros)	(N-r)/(rc-r-X)	42.86%

Figure S6. GROOVE analysis of overlap in studies on ESD-induced ulcer healing and rebamipide use

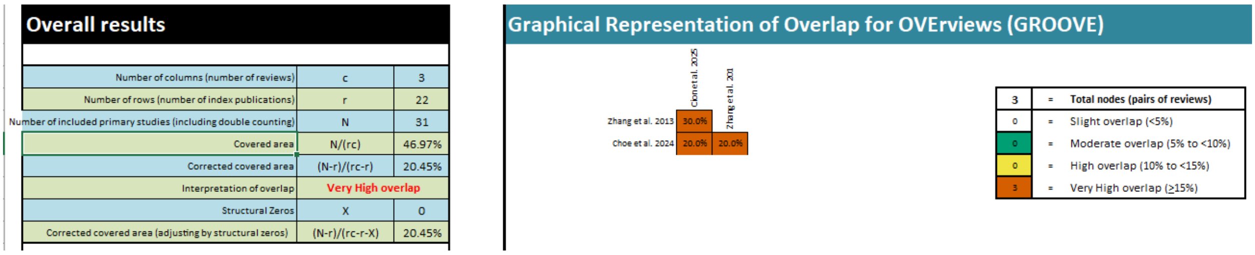

Overall results

Number of columns (number of reviews)	c	3
Number of rows (number of index publications)	r	22
Number of included primary studies (including double counting)	N	31
Covered area	N/(rc)	46.97%
Corrected covered area	(N-r)/(rc-r)	20.45%
Interpretation of overlap	Very High overlap	
Structural Zeros	X	0
Corrected covered area (adjusting by structural zeros)	(N-r)/(rc-r-X)	20.45%

Figure S7. GROOVE analysis of overlap in studies on NSAID-induced ulcers and rebamipide use

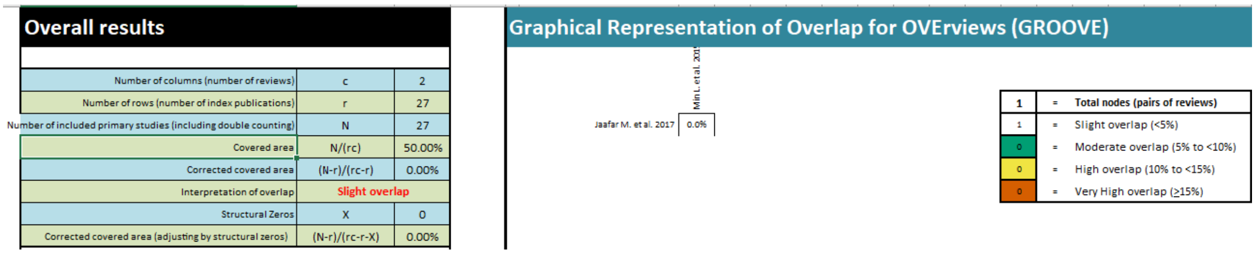

Overall results		
Number of columns (number of reviews)	c	2
Number of rows (number of index publications)	r	27
Number of included primary studies (including double counting)	N	27
Covered area	N/(rc)	50.00%
Corrected covered area	(N-r)/(rc-r)	0.00%
Interpretation of overlap	Slight overlap	
Structural Zeros	X	0
Corrected covered area (adjusting by structural zeros)	(N-r)/(rc-r-X)	0.00%

Figure S8. GROOVE analysis of overlap in studies on dyspeptic symptoms and rebamipide use

Section S3. Egger's test

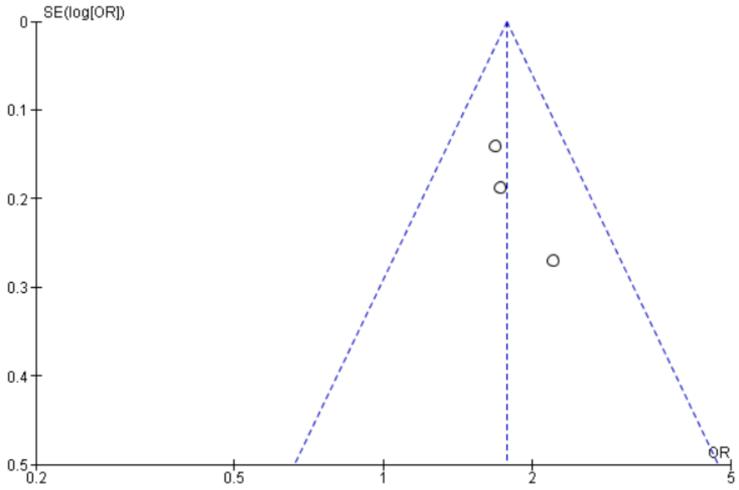

Figure S9. Funnel plot for Odds ratio for *H.pylori* eradication rates in regimens including rebamipide versus standard therapy.

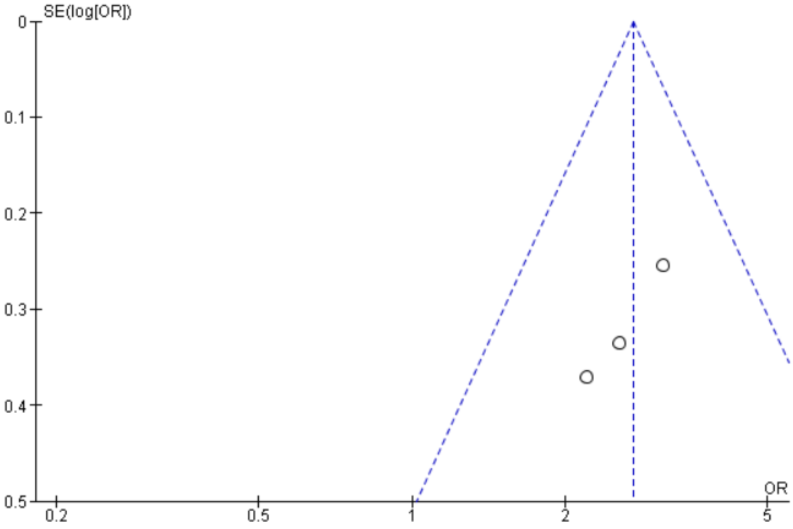

Figure S10. Funnel plot for Effect of Therapy in Patients Receiving Rebamipide vs Controls During NSAID Therapy

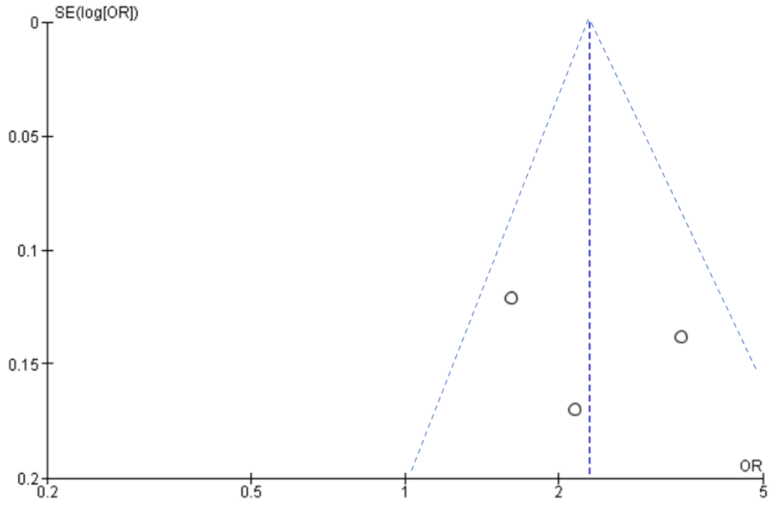

Figure S11. Funnel plot for Odds ratio for the effect of adding rebamipide to PPI therapy on ESD-induced ulcer healing.

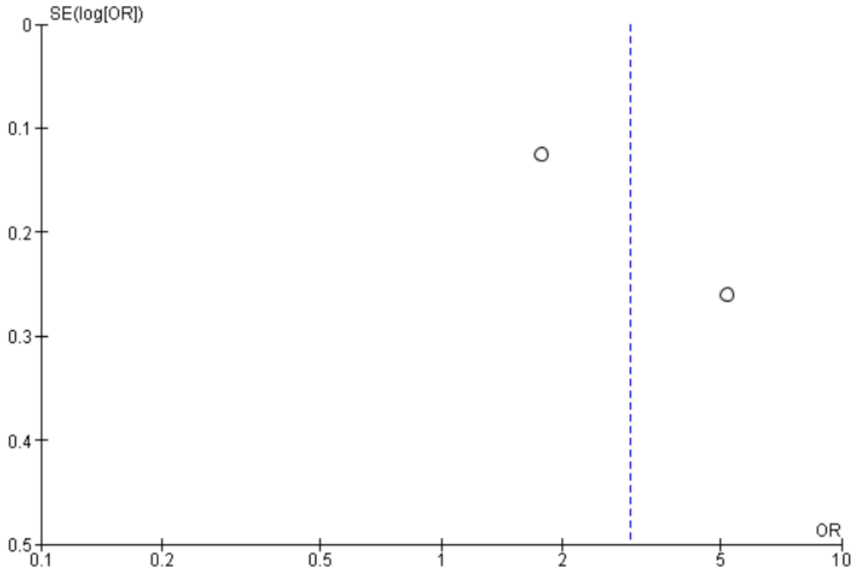

Figure S12. Funnel plot for Odds ratio for the effect of adding rebamipide to dyspeptic symptoms treatment.
